# The economic and environmental effects of the Beijing-Tianjin-Hebei Collaborative Development Strategy— taking Hebei Province as an example

**DOI:** 10.1007/s11356-020-09790-1

**Published:** 2020-06-29

**Authors:** Rongxia Zhang, Suocheng Dong, Zehong Li

**Affiliations:** 1 Institute of Geographic Sciences and Natural Resources Research, Chinese Academy of Sciences, Beijing, 100101 China; 2 School of Economics and Management, Taiyuan University of Technology, Taiyuan, 030024 China

**Keywords:** Beijing-Tianjin-Hebei Collaborative Development Strategy, Counterfactual analysis, Treatment effects, Panel data approach

## Abstract

In 2014, the Beijing-Tianjin-Hebei Collaborative Development Strategy (hereinafter the Jing-Jin-Ji Strategy) was formally proposed as a major national strategy, providing an unprecedented opportunity for the overall development of Hebei. This article evaluates the treatment effects of the Jing-Jin-Ji Strategy on Hebei’s economy and environment. Employing a panel data program evaluation method developed by Hsiao et al. (2012), we construct hypothetical counterfactuals for the GDP growth rate, the percentage of tertiary industry in GDP, and the geographic mean PM_2.5_ concentrations for Hebei in the absence of the Jing-Jin-Ji Strategy using the outcomes of selected untreated provinces. The results show that the Jing-Jin-Ji Strategy increased the percentage of tertiary industry in GDP by an average of 2.53 percentage points per year between 2014 and 2018 and decreased the geographic mean PM_2.5_ concentrations by an average of 11.1 percentage points per year between 2014 and 2017. However, it does not appear to have had significant effects on Hebei’s GDP growth rate. The leave-one-out method demonstrates the robustness of the above results. This article suggests that Hebei should speed up its economic growth and bridge the gap with Beijing and Tianjin while ensuring the quality of its economic development and a sound ecological environment.

## Introduction

At present, China has three world-class urban agglomerations: the Yangtze River Delta, the Pearl River Delta, and the Beijing-Tianjin-Hebei (hereinafter Jing-Jin-Ji) agglomeration. These are the core embodiment of the development level and competitiveness of China (Liu et al. 2020). Among them, the Jing-Jin-Ji agglomeration, as the political center and third largest economy in China (Xie et al. 2017), has received an increasing social attention. This region consists of two municipalities (Beijing and Tianjin) and one province (Hebei), accounting for 2.2% of China’s land. Data from 2018 show that this region accounts for 8.1% of China’s population and yields 9.3% of its total GDP (China Statistical Yearbook, 2019). However, due to excessive development, the region faces two severe problems. First, the Jing-Jin-Ji region is the most unbalanced of the three urban agglomerations in terms of economic development (Gao et al. 2017); second, the Jing-Jin-Ji region is one of the most atmospherically polluted regions in China (Li et al. 2019a), and five of the ten cities at the bottom of China’s air quality rankings for 2018 belong to the Jing-Jin-Ji region according to the 2018 China Ecological Environment Bulletin. Regional development imbalance and pollution are major constraints on economic development (Mohsin et al. 2019), and thus these problems have seriously hindered the healthy development of the Jing-Jin-Ji region. Therefore, the challenge facing the Jing-Jin-Ji region is how to improve the quality of economic growth under environmental constraints, aiming to achieve a win-win situation for the economic development and environmental protection. Thus, the Beijing-Tianjin-Hebei Collaborative Development Strategy (hereinafter the Jing-Jin-Ji Strategy) was formally proposed in 2014 as a national strategy. It aims to build an integrated Jing-Jin-Ji region with higher-quality economic growth, a more reasonable industrial structure, and a better ecological environment (Li et al. 2019c). Therefore, the Jing-Jin-Ji Strategy aims to effectively alleviate the current problems facing the Jing-Jin-Ji region.

With the implementation of the Jing-Jin-Ji Strategy, the central and local governments have issued a series of policies, e.g., *The Implementing Rules of Air Pollution Prevention and Control Action Plan in the Jing-Jin-Ji Region and Surrounding Provinces*, *The Guideline for the Collaborative Development of the Jing-Jin-Ji Region*, *The Guideline for Industrial Transfer in the Jing-Jin-Ji Region*, and *The Ecological and Environmental Protection Plan for the Jing-Jin-Ji Collaborative Development*. To date, these policies have made great achievements in promoting economic development and reducing environmental pollution, as addressed in some previous published studies. Most of the literature examines the effects of a particular policy issued within the framework of the Jing-Jin-Ji Strategy (Liu et al. 2020; Zhang et al. 2019; Tian et al. 2019; Song et al. 2020; Huang et al. 2018; Feng et al. 2019), but except for Dai (2019), few studies examine the effects of the Jing-Jin-Ji Strategy as a whole. We believe that these policies were released within the framework of the Jing-Jin-Ji Strategy, so their effects as a whole should be regarded as the overall effect of the Jing-Jin-Ji Strategy. To better understand the effects of policy implementation and provide evidence for future policy adjustments, we conduct a quantitative policy evaluation of the Jing-Jin-Ji Strategy. Although studies have provided valuable insights into the treatment effects of the Jing-Jin-Ji Strategy, few apply a counterfactual framework to policy analysis for the Jing-Jin-Ji Strategy, with the exception of Dai et al.’s (2019) case study. Although Dai (2019) and our research are both under a counterfactual framework, Dai (2019) apply the synthetic control method (SCM) (Abadie and Gardeazabal 2003; Abadie et al. 2010), while our research applies the panel data approach (PDA) (Hsiao et al. 2012). The PDA and the SCM are the only program evaluation methodologies that can handle estimation of the treatment effect on a single unit, whereas other methodologies require more than one treated unit (Gardeazabal and Vega-Bayo 2017). Both the PDA and the SCM are measurement-without-theory approaches that construct counterfactuals by exploiting the correlations among cross-sectional units and do not need complicated economic models. The primary differences between these two approaches are as follows: (1) the PDA estimates the weights of the control units by ordinary least squares (OLS), but the SCM estimates the weights with covariates. (2) The SCM constrains the weights of the units in the control group to be nonnegative and requires that they add up to one, so extrapolation outside the convex hull of the covariates for the treated units is not allowed (Gardeazabal and Vega-Bayo 2017). However, Wan et al. (2018) conclude that the SCM convex hull constraints are not needed or necessarily satisfied in many cases. The PDA does not place any restriction on the weights of the units and includes an intercept to take into account the differences in individual-specific fixed effects between the treated and control units, which are critical for generating unbiased predictions of the counterfactuals and make more economic sense (Wan et al. 2018). (3) The simulation designs by Wan et al. (2018) also show that the PDA dominates the SCM in many cases when measured by the mean square error. Therefore, we will adopt the PDA to analyze the economic and environmental effects of the Jing-Jin-Ji Strategy. In addition to the research methods, Dai (2019) estimated the economic effects of the Jing-Jin-Ji Strategy from 2001 to 2014 because they thought efforts at collaboration between Beijing, Tianjin, and Hebei gained new impetus in 2001; however, we estimate the economic effects from 2014 to 2018 and the environmental effects from 2014 to 2017 because we believe the Jing-Jin-Ji Strategy only began to play a substantial role after it was proposed as a national strategy in 2014.

As the core cities of this agglomeration, for many years, Beijing and Tianjin have attracted a large amount of high-quality resources from Hebei while having a much weaker diffusion effect on the province; thus, Hebei has lagged far behind Beijing and Tianjin in terms of development speed and quality. The Jing-Jin-Ji Strategy is an unprecedented opportunity for Hebei, as it provides Hebei with various high-quality resources, such as strong scientific and technological support and talent. In this case, compared with Beijing and Tianjin, we believe that the Jing-Jin-Ji Strategy has a greater effect on Hebei because the overall strength of Hebei is much weaker. Moreover, within the sample period, Hebei is treated mainly by the Jing-Jin-Ji Strategy. However, Beijing, as the capital, is treated by many small- and medium-sized policies in addition to the Jing-Jin-Ji Strategy, such as the National Tourism Comprehensive Reform Pilot City implemented in 2012. Similarly, Tianjin is also treated by the China (Tianjin) Pilot Free Trade Zone since 2015. When the treatment effects of multiple policies are mixed together, it is difficult to distinguish the effects of one policy from another. Therefore, we will take Hebei as an example to estimate the Jing-Jin-Ji Strategy’s economic and environmental effects under the counterfactual framework.

The rest of the article is organized as follows. Section 2 describes the methodological framework of Hsiao et al. (2012). Section 3 introduces the data and settings required to implement the method. Section 4 presents the economic and environmental effects of the Jing-Jin-Ji Strategy. Section 5 reports the robustness tests of the treatment effects. Section 6 presents the conclusion and policy implications.

## Methodological framework

### The counterfactual framework

At present, the construction of counterfactuals to evaluate the effects of a policy is a research focus for policy evaluation. Many scholars in China and elsewhere have theoretical and applied achievements of great significance in counterfactual analysis.

Suppose that we have *N* provinces, which can be divided into two categories. The provinces treated or receiving intervention by the Jing-Jin-Ji Strategy constitute the treatment group. The provinces untreated by the Jing-Jin-Ji Strategy constitute the control group. Let ${y}_{it}^1$ and ${y}_{it}^0$ denote the outcomes of the *i*th province with and without the Jing-Jin-Ji Strategy in year *t*, respectively. For years *1* to *T*_1_, there is no policy treatment, so ${y}_{it}^1$=${y}_{it}^0,t=1,\dots, {T}_1$. From year *T*_1_ + 1, some provinces will be under treatment. For provinces under treatment, we can only observe ${y}_{it}^1\ \mathrm{for}\ t={T}_1+1,\dots, T$, which refers to what has occurred with the Jing-Jin-Ji Strategy, and cannot observe ${y}_{it}^0$, which is what would have occurred in the absence of the Jing-Jin-Ji Strategy. For provinces without treatment, we can only observe ${y}_{it}^0,t={T}_1+1,\dots, T$, but not ${y}_{it}^1$.

Then, the treatment effect of the Jing-Jin-Ji Strategy for the *i*th province under treatment in year *t* is as follows:

1 $$ {\Delta }_{\mathrm{i}t}={y}_{\mathrm{i}t}^1-{y}_{\mathrm{i}t}^0\kern0.5em \mathrm{t}={T}_1+1,\dots, \mathrm{T}, $$

Hebei is the only province in the treatment group in our case. Without loss of generality, we assume that *i* = 1 represents Hebei and that *i* = 2, …, *N* represents the provinces in the control group. Hebei is treated by the Jing-Jin-Ji Strategy, so ${y}_{1t}^1$ is observable. ${y}_{1t}^0$ is the ex post counterfactual, which is unobservable and should be constructed with an econometric model. The Jing-Jin-Ji Strategy is obviously not a randomized control trial, so counterfactuals can be constructed with quasi-experimental methods, e.g., regression discontinuity design (RDD), the instrumental variables (IV) method, and propensity score matching with the difference-in-difference (PSM-DID) regression approach (Heckman and Vytlacil 2007; Imbens and Wooldridge 2009; Pelucha et al. 2019). However, these methods are based on certain assumptions, sufficient data, and reasonable economic models (Zhang et al. 2016). In reality, the mechanisms of the Jing-Jin-Ji Strategy influencing Hebei’s economy and environment are too complicated to be depicted by economic models, and we only have finite panel data, so the above approaches are inappropriate. In addition to the above methods, which are based on measurement with theory, some measurement-without-theory approaches have been widely applied, one of which is the PDA developed by Hsiao et al. (2012). The PDA can overcome the problems faced by methods based on measurement with theory; we will next give a brief introduction to the PDA.

### The panel data approach

Based on the panel data, the PDA assumes that ${y}_{it}^0$ is generated by a factor model:

2 $$ {y}_{it}^0={\alpha}_i+{b}_i^{\prime }{f}_t+{\varepsilon}_{it}\kern0.5em i=1,\dots, \kern0.5em {\displaystyle \begin{array}{cc}N;t=1,\dots, & T\end{array}} $$

where *α*_*i*_ denotes the fixed effects, ${b}_i^{\prime }$ denotes the *1 × K* vector of constants, *f*_*t*_ denotes the *K × 1* common factors that drive all cross-sectional units, and *ε*_*it*_ denotes the idiosyncratic error with E(*ε*_*it*_) = 0.

For convenience, Eq. 2 can be expressed by the following matrix equation:

3 $$ {y}_t^0=\alpha +B{f}_t+{\varepsilon}_t\kern0.5em i=1,\dots, \kern0.5em {\displaystyle \begin{array}{ccc}N&\ t=1,\dots, & T\end{array}} $$

where ${y}_t^0=\Big({y}_{1t}^0,\dots,^{\prime },\alpha ={\left(\ {\alpha}_1,\dots, {\alpha}_N\right)}^{\prime },\kern0.5em {\varepsilon}_t={\left({\varepsilon}_{1t},\dots, {\varepsilon}_{Nt}\right)}^{\prime }$ and *B* = (*b*_1_, …, b_*N*_)^′^ is the *N × K* factor loading matrix.

When Eq. 3 satisfies the following assumptions (1–5)—(1) ‖*b*_*i*_‖ = *c* <  ∞ for *i* = 1, …, *N*; (2) *ε*_*t*_ is *I(0)* with E(*ε*_*t*_) = 0, and $\mathrm{E}\left({\varepsilon}_t{\varepsilon}_t^{\prime}\right)=V$, where *V* is a diagonal constant matrix; (3)$E\left({\varepsilon}_t{f}_t^{\prime}\right)$*=0*; (4) *R(B) = K*; and (5) *E*(*ε*_*js*_| *d*_*it*_) = 0 *for j* ≠ *i*—if both *N* and *T* are large enough, the method of Bai and Ng (2002) can be used to identify the number of common factors, *K*. In addition, *α*, *B and f*_*t*_ can be estimated by the maximum likelihood approach. Then, the ex post counterfactual of Hebei, ${y}_{1t}^0$, can be predicted by ${\hat{y}}_{1t}^0={\hat{\alpha}}_1+{{\hat{b}}_1}^{\prime }{\hat{f}}_t$ for *t*= *T*_1_ + 1,…, *T*. However, for macroeconomic data, usually neither *N* nor *T* is large. In this case, Hsiao et al. (2012) attribute the cross-sectional dependence to the presence of common factors that drive all the relevant cross-sectional units. Based on this premise, they prove that as long as assumption (5) *E*(*ε*_*js*_| *d*_*it*_) = 0 *for j* ≠ *i* holds, the outcomes of the untreated units (*y*_2*t*_, …, *y*_*Nt*_) can be used to predict ${y}_{1t}^0$ instead of identifying *α*_1_,*b*_1_ and *f*_*t*_ (e.g., Ching et al. 2012; Du and Zhang 2015), that is,

4 $$ {\hat{y}}_{1t}^0=c+{\hat{\beta}}^{\prime }\tilde{y}_{t}\kern0.5em t={T}_1+1,\dots, \kern0.5em T $$

where $$ \tilde{y}_{t}={\left({y}_{2t},\dots, {y}_{Nt}\right)}^{\prime } $$.

Then,

5 $$ {\hat{\Delta }}_{1t}={y}_{1t}-{\hat{y}}_{1t}^0\kern0.5em t={T}_1+1,\dots, \kern0.5em T $$

The next issue is how to choose the best predictive model to construct counterfactuals. Hsiao et al. (2012) prove that when *T*_1_ → ∞, the within-sample fit increases with cross-sectional units. However, on many occasions, *T*_1_ is finite. As more cross-sectional units are used, the variance of $\hat{\beta}$ will also increase, which will lead to inaccurate post-sample prediction. To balance the within-sample fit with the post-sample prediction accuracy, Hsiao and Wan (2014) suggest using the 2-step method to select the best predictive model. Step 1: Use *R*^2^ to select the best predictors for ${\hat{y}}_{1t}^0$ using *j* units out of the *N − 1* units without treatment, denoted by *M*(*j*)^∗^, for *j* = *1*, …, *N − 1*. Step 2: Choose *M*(*m*)^∗^ from *M*(1)^∗^, *M*(2)^∗^,…, *M*(*N* − 1)^∗^ in terms of the corrected Akaike information criterion (AICC).

### The basic steps of PDA

From the above analysis, the basic steps of PDA can be summarized as follows (Mao 2018):

1. Identify the outcome variables and the treatment or intervention according to the research question.
2. Identify a treated unit and a set of control units.
3. Perform step 1 of the 2-step method to obtain *M*(*j*)^∗^ (j = 1, …, N − 1) based on the data of the preintervention period.
4. Perform step 2 of the 2-step method to obtain *M*(*m*)^∗^; thus, the predictive model is obtained.
5. Predict the counterfactual ${\hat{y}}_{1t}^0$ in the postintervention period.
6. Estimate the treatment effects according to Eq. 5.
7. Check the statistical significance of the treatment effects.
8. Use leave-one-out tests to check the robustness of the estimated treatment effects.

The PDA is particularly suitable for the macroeconometric context (Bai et al. 2014; Bove et al. 2016; Wan et al. 2018), and it has been applied to estimate the macroeconomic effects of China’s entry to the WTO (Ching et al. 2011), the policy initiatives launched by Japan’s Prime Minister Shinzo Abe in his first quarter in the office (Hayashi 2014), China’s 2008 economic stimulus package (Ouyang and Peng 2015), China’s high-speed rail projects (Ke et al. 2017), and China’s Clean Air Action (Li et al. 2019b).

## Data and settings

In this section, we will introduce the data and settings for properly implementing the PDA.

### Data

In this study, the GDP growth rate, percentage of tertiary industry in GDP, and geographic mean PM_2.5_ concentrations are employed to represent economic growth, industrial structure, and environmental pollution, respectively. Provinces are our primary unit of study. To apply PDA, we need panel data on the annual GDP growth rate, percentage of tertiary industry in GDP, and geographic mean PM_2.5_ concentrations across provinces, including Hebei and the control groups. Based on data availability, the sample periods for the GDP growth rate, percentage of tertiary industry in GDP, and geographic mean PM_2.5_ concentrations are 1990–2018, 1992–2018, and 2000–2017, respectively. The preintervention and postintervention periods for each indicator are shown in Table 1. The data of GDP growth rate and percentage of tertiary industry in GDP are from the China Statistical Yearbook and those of geographic mean PM_2.5_ concentrations are from the Atmosphere Composition Analysis Group of Dalhousie University in Canada.

**Table 1:** Data and settings

Outcome variables	GDP growth rate	Percentage of tertiary industry in GDP	Geographic mean PM_2.5_ concentrations
Treatment group units	Hebei	Hebei	Hebei
Control group units	15 provinces	19 provinces	18 provinces
Preintervention period	1990–2013	1992–2013	2000–2013
Postintervention period	2014–2018	2014–2018	2014–2017

### Settings

#### Treatment group choice

In this study, the only unit in the treatment group was Hebei. The aim of our study is to estimate Hebei’s counterfactuals for the above three indicators, and the effects of the Jing-Jin-Ji Strategy are the differences between the actual values and the counterfactuals.

#### Control group choice

The provinces in the control group should satisfy two criteria. The strictest criterion is that each province in the control group should be exogenous to the policy treatment, as indicated by assumption 5; that is, to avoid endogeneity of the explanatory variables, the provinces in the control group should not be influenced by the Jing-Jin-Ji Strategy. To ensure that assumption 5 is not violated, we should exclude Beijing and Tianjin because they are under the treatment of the Jing-Jin-Ji Strategy. Then, following the idea of Kline and Moretti (2014), we do not include any untreated provinces that border Beijing, Tianjin, or Hebei in our control group because geographically proximate provinces may benefit from the Jing-Jin-Ji Strategy. The second criterion is that the provinces in the control group should be good predictors for the outcomes of Hebei before the intervention of the Jing-Jin-Ji Strategy. Accordingly, for each of the three indicators mentioned above, we obtained 15, 19, and 18 provinces^1^ satisfying the exogeneity criterion that also had consistently reported data during our research period. These provinces are geographically distant from Hebei, which is fully permissible when applying PDA to construct counterfactuals because PDA’s basic idea is to select the best predictors rather than finding a control group that is similar in covariates with the treatment group.

## Treatment effects of the Jing-Jin-Ji Strategy

### The estimation

According to Hsiao et al. (2012), if the reactions of the provinces to the Jing-Jin-Ji Strategy are similar or even if their responses are different, as long as (*y*_1*t*_, *y*_2*t*_, …, *y*_*Nt*_) are driven by some common factors, information on the provinces without treatment can help to construct the counterfactuals of the treated province. In our case, each province must be driven by some common factors since all the provinces are in China. Therefore, the treatment effects of the Jing-Jin-Ji Strategy can be estimated by the PDA. By experimenting with different combinations of provinces in the control group following the 2-step method described in Section 2.2, we found the provinces that could best balance preintervention fit with postintervention prediction accuracy and used them to construct counterfactuals. The selected provinces and the OLS estimates of the weights in the predictive models are listed in Tables 2, 3, and 4. Tables 2, 3, and 4 show that all three indicators have good within-sample fit, with an *R*-square above 0.93 and an *F*-statistic above 50. These results indicate that the provinces selected according to the AICC perform well, so the actual values and their predicted counterfactuals are comparable in the postintervention period.

**Table 2:** Weights of the control group for the GDP growth rate

	Beta	Std	T
Constant	0.491	0.942	0.52
Zhejiang	0.393	0.082	4.82
Fujian	0.380	0.117	3.25
Guangxi	−0.396	0.134	−2.95
Sichuan	0.515	0.114	4.52

*R*^2^ = 0.934, *AICC* = 3.8942, *F* = 50.273

**Table 3:** Weights of the control group for the percentage of tertiary industry in GDP

	Beta	Std	T
Constant	6.746	1.019	6.62
Heilongjiang	0.178	0.037	4.77
Shanghai	−0.302	0.044	−6.87
Jiangsu	0.256	0.049	5.24
Anhui	−0.169	0.030	−5.65
Fujian	0.356	0.041	8.69
Guangdong	0.256	0.039	6.51
Guangxi	0.275	0.035	7.77
Guizhou	0.114	0.020	5.60
Yunnan	−0.170	0.041	−4.17

*R*^2^ = 0.996, *AICC* = −48.475, *F* = 352.95

**Table 4:** Weights of the control group for the geographic mean PM_2.5_ concentrations

	Beta	Std	T
Constant	1.283	4.317	0.30
Fujian	4.792	0.351	13.63
Guangdong	−2.495	0.338	−7.38
Guangxi	1.892	0.183	10.34
Jilin	1.656	0.122	13.52
Jiangxi	−1.604	0.238	−6.75
Zhejiang	−0.682	0.207	−3.30

*R*^2^ = 0.990, *AICC* = 36.9868, *F* = 110.36

**Fig. 1:**
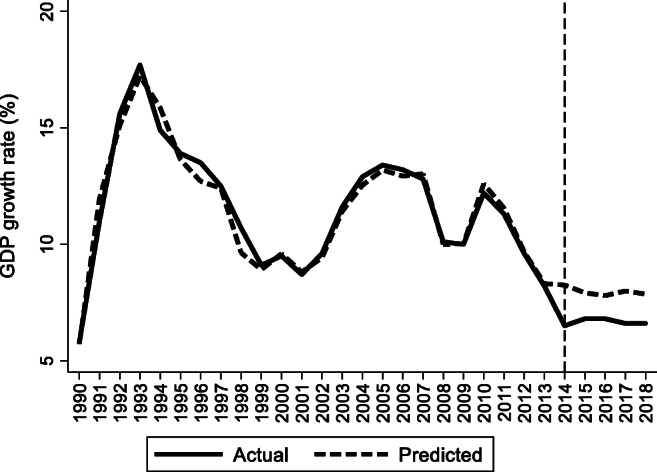
Actual and predicted paths of the GDP growth rate: the whole sample (The vertical line represents the year of implementation for the Jing-Jin-Ji Strategy.)

**Fig. 2:**
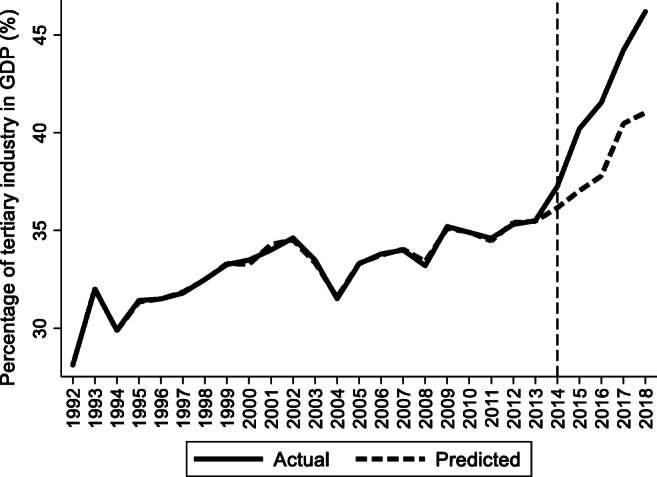
Actual and predicted paths of the percentage of tertiary industry in GDP: the whole sample

**Fig. 3:**
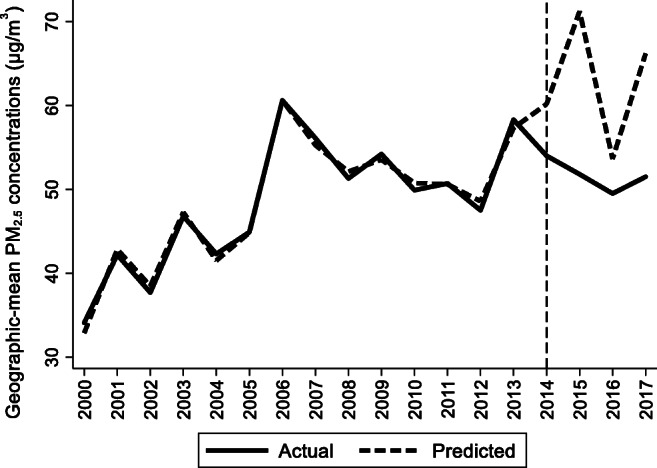
Actual and predicted paths of the geographic mean PM_2.5_ concentrations: the whole sample

**Fig. 4:**
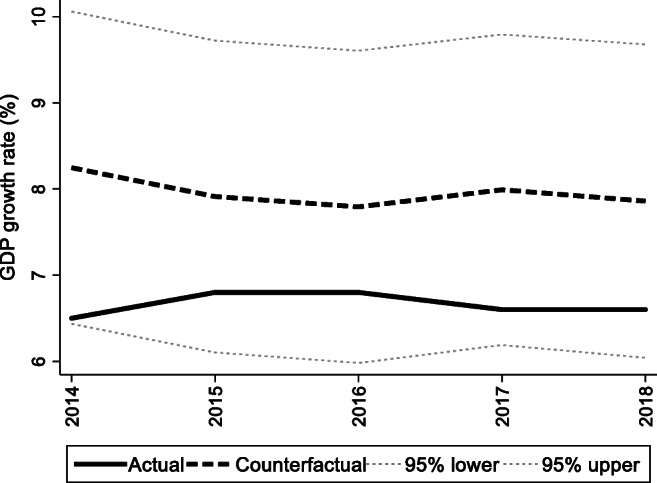
Actual and counterfactual paths of the GDP growth rate: the postintervention period

**Fig. 5:**
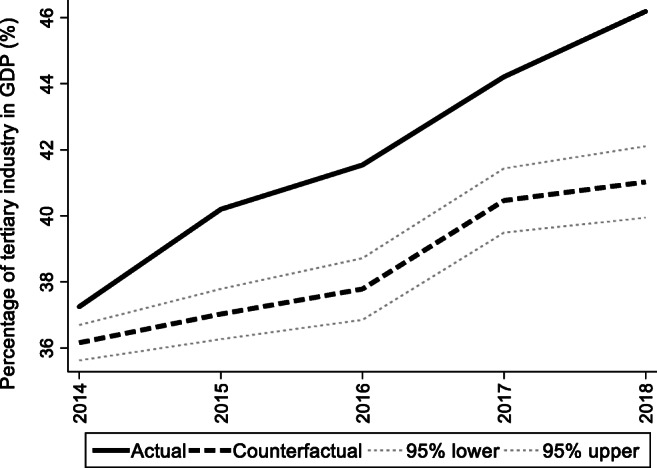
Actual and counterfactual paths of the percentage of tertiary industry in GDP: the postintervention period

**Fig. 6:**
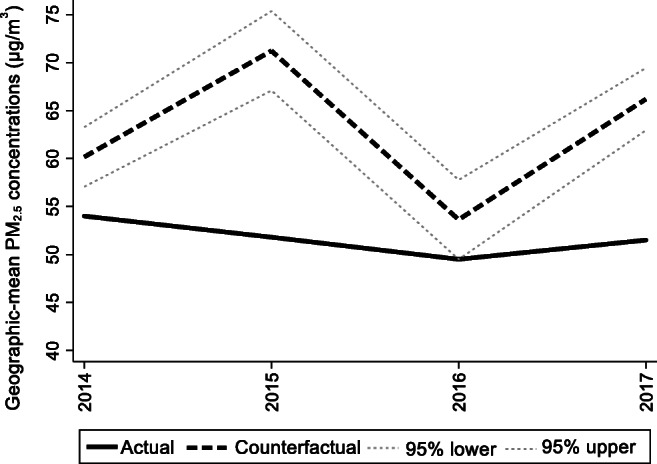
Actual and counterfactual paths of the geographic mean PM_2.5_ concentrations: the postintervention period

**Fig. 7:**
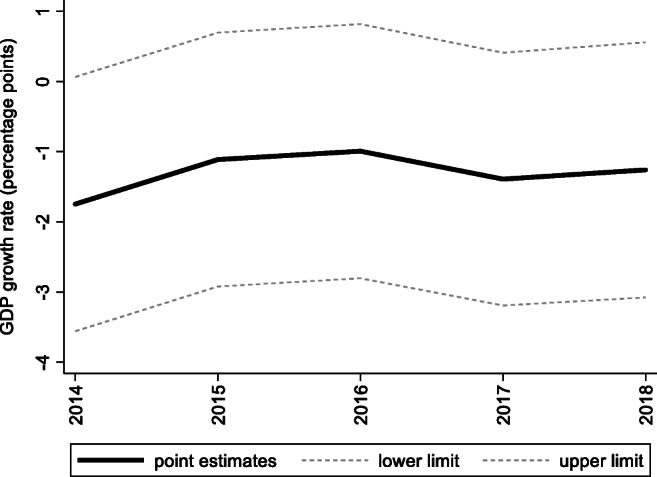
Treatment effects for the GDP growth rate

**Fig. 8:**
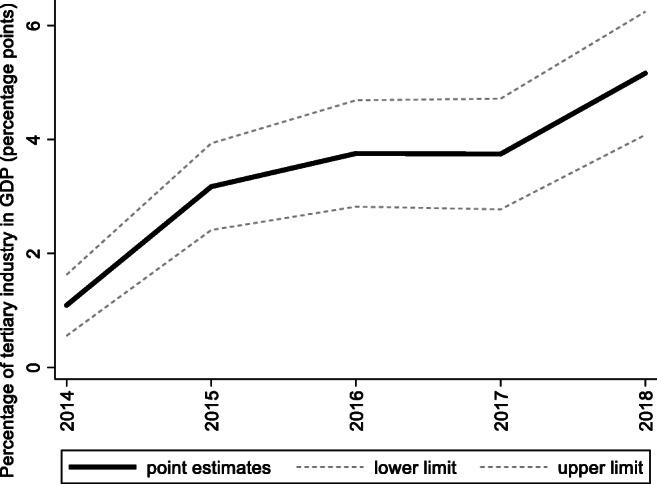
Treatment effects for the percentage of tertiary industry in GDP

**Fig. 9:**
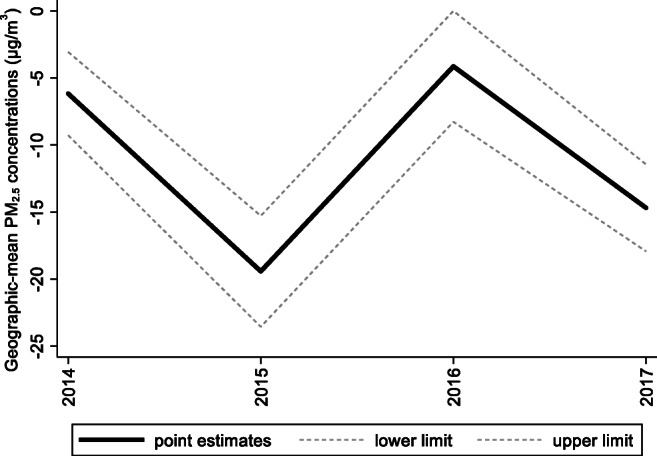
Treatment effects for the geographic mean PM_2.5_ concentrations

For the preintervention period, Tables 2, 3, and 4 list the provinces selected from the control groups by the AICC that could be used to construct the hypothetical paths for the three indicators of Hebei. Therefore, the ${y}_{1t}^0$ of Hebei is a linear combination of the selected provinces with either positive or negative weights. Hence, for each of the three indicators of Hebei, the selected provinces from the control group combined play a role in constructing the counterfactual path. Based on Tables 2, 3, and 4, we plot the predicted paths of the three indicators for the whole sample period, as shown by the dashed lines in Figs. 1, 2, and 3. The solid lines in Figs. 1, 2, and 3 are the actual paths of the three indicators for the whole sample period. In Figs. 1, 2, and 3, during the preintervention period, the predicted paths fit the actual paths well, and during the postintervention period, the gaps between the actual paths and the predicted paths are the effects of the Jing-Jin-Ji Strategy. The predicted path in the postintervention period can also be called the counterfactual path. Figures 4, 5, and 6 plot the actual and counterfactual paths during the postintervention period, with the gray dashed lines denoting the 95% confidence intervals of the counterfactual paths. Following Hsiao (2014), the point estimates and interval estimates of the treatment effects are plotted directly in Figs. 7, 8, and 9. In addition to the graphical representation of the treatment effects, specific values for point and interval estimates are given in Tables 5, 6, and 7.

**Table 5:** Treatment effects for the GDP growth rate

Year	Actual	Counterfactual	Treatment effects	
			Point	Interval
2014	6.5	8.25	−1.75	(−3.56, 0.07)
2015	6.8	7.91	−1.11	(−2.92, 0.70)
2016	6.8	7.79	−0.99	(−2.80, 0.82)
2017	6.6	7.99	−1.39	(−3.19, 0.41)
2018	6.6	7.86	−1.26	(−3.08, 0.56)

**Table 6:** Treatment effects for the percentage of tertiary industry in GDP

Year	Actual	Counterfactual	Treatment effects	
			Point	Interval
2014	37.25	36.16	1.09	(0.55, 1.63)
2015	40.20	37.03	3.17	(2.41, 3.93)
2016	41.54	37.78	3.76	(2.82, 4.69)
2017	44.21	40.46	3.75	(2.77, 4.72)
2018	46.19	41.03	5.16	(4.08, 6.25)

**Table 7:** Treatment effects for the geographic mean PM_2.5_ concentrations

Year	Actual	Counterfactual	Treatment effects	
			Point	Interval
2014	54.00	60.16	−6.16	(−9.27, −3.06)
2015	51.80	71.22	−19.42	(−23.55, −15.28)
2016	49.50	53.63	−4.13	(−8.27, 0)
2017	51.5	66.18	−14.68	(−17.93, −11.44)

### Empirical results

For Hebei’s GDP growth rate, as shown in Fig. 4, the counterfactual path is higher than the actual path, so the effects are negative. By calculating the 95% confidence interval of the predicted counterfactuals, we find that the negative treatment effects are not statistically significant at the 5% level because the actual path is within the upper and lower limits of the estimated confidence interval of the counterfactual path. The same result can be obtained from Fig. 7 because the confidence interval for the point estimates of the treatment effects for each of the 4 years includes zero, which also indicates that the treatment effects are not statistically significant. Table 5 shows that the counterfactuals of the GDP growth rate in Hebei from 2014 to 2018 are 8.25, 7.91, 7.79, 7.99, and 7.86%, respectively. The point estimates of the treatment effects in Table 5 are −1.75, −1.11, −0.99, −1.39, and −1.26 for 2014–2018, respectively, which means that the GDP growth rate of Hebei would be 1.75, 1.11, 0.99, 1.39, and 1.25 percentage points higher without the Jing-Jin-Ji Strategy, respectively, but the results are not statistically significant. Table 5 also reports the interval estimates. Because a point estimate does not tell us how close the estimate is likely to be to the parameter, an interval estimate incorporates a margin of error that helps us gauge the accuracy of the point estimate and enables us to see both how small and how large an effect may be. The interval estimates suggest that even if Hebei’s GDP growth rate was equal to the upper limit of the interval estimates, the Jing-Jin-Ji Strategy could only increase Hebei’s GDP growth rate by 0.51 percentage points per year on average.

For Hebei’s percentage of tertiary industry in GDP, Fig. 5 shows that the treatment effects are positive. Meanwhile, the upper 95% confidence limit of the counterfactual path in Fig. 5 is much lower than the actual path, and the confidence intervals for the treatment effects in Fig. 6 do not contain zero, so the positive effects are statistically significant at the 5% level. The point and interval estimates are reported in Table 6. The point estimates of the treatment effects in Table 6 are 1.09, 3.17, 3.76, 3.75, and 5.16 for 2014–2018, respectively, which means that the percentage of tertiary industry in GDP would be 1.09, 3.17, 3.76, 3.75, and 5.16 percentage points lower without the Jing-Jin-Ji Strategy. Therefore, the average percentage of tertiary industry in GDP increased by an average of 3.39 percentage points per year between 2014 and 2018. Even if the annual increase in the percentage of tertiary industry in GDP equals the lower limit of the interval estimates, it grows by an average of 2.53 percentage points per year between 2014 and 2018, which is a considerable increase.

For Hebei’s geographic mean PM_2.5_ concentrations, the treatment effects are negative, as shown in Fig. 6. By calculating the 95% confidence interval of the predicted counterfactuals in Fig. 6 and the treatment effects in Fig. 9, we find that the negative treatment effects are statistically significant. The point estimates of the treatment effects are −6.16, −19.42, −4.13, and −14.68 μg/m^3^, as shown in Table 7. Therefore, we may conclude that the Jing-Jin-Ji Strategy has significantly reduced Hebei’s geographic mean PM_2.5_ concentrations. Without the Jing-Jin-Ji Strategy, the values of geographic mean PM_2.5_ concentrations would be 6.16, 19.42, 4.13, and 14.68 μg/m^3^ higher than the actual values for 2014–2017, respectively. On average, the geographic mean of PM_2.5_ in Hebei between 2014 and 2017 decreased by 11.1 percentage points annually due to the implementation of the Jing-Jin-Ji Strategy. The upper limits of the counterfactuals’ interval estimates are less than 0 except for 2016, when it equals 0. Therefore, the least desirable treatment effect of the Jing-Jin-Ji Strategy on geographic mean PM_2.5_ concentrations of Hebei equals 0. If the geographic mean PM_2.5_ concentrations in Hebei are reduced by the upper limits of the interval estimates (the worst-case scenario), the annual average reduction is 7.45 percentage points between 2014 and 2017.

## Robustness tests

To evaluate the credibility of treatment effects in Tables 5, 6, and 7, we cannot resort to the GDP growth rate, percentage of tertiary industry in GDP, and geographic mean PM_2.5_ concentrations of Hebei in the absence of the Jing-Jin-Ji Strategy, since the counterfactuals are unobservable. We note that there are two critical assumptions underlying this approach. (1) The impacts of the Jing-Jin-Ji Strategy should be local, so the provinces in the control group should not be affected by the Jing-Jin-Ji Strategy. (2) All the provinces in the treatment and control groups should be driven by the same common factors, and the fundamental relations between the treated provinces and the untreated provinces before treatment should remain unchanged in the absence of treatment in the postintervention period. If these two assumptions hold, then our results are reliable. However, a direct test for these two assumptions is infeasible because there are no observations of the outcomes of the treated provinces in the absence of the Jing-Jin-Ji Strategy. We will thus resort to an indirect method to justify our estimates in this section. We adopt the leave-one-out robustness test, which has been used in Abadie et al. (2015), and the estimated effect is robust only if it is insensitive to the leave-one-out design (Mao 2018).

**Fig. 10:**
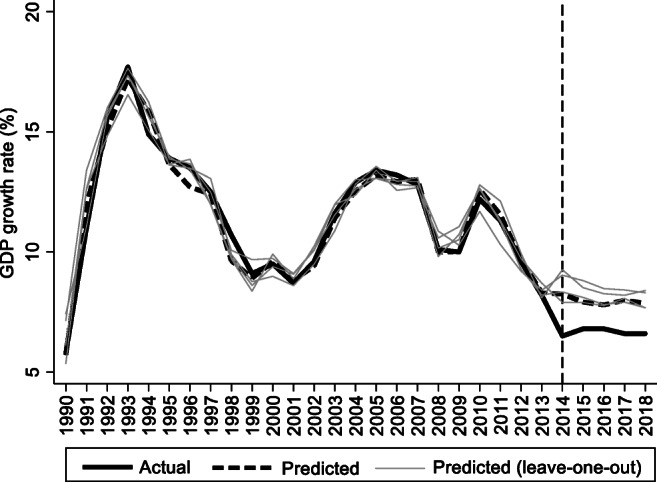
Leave-one-out distribution for the GDP growth rate

For Hebei’s economic growth rate, we recall from Table 2 that the predicted path is estimated as a weighted average of Zhejiang, Fujian, Guangxi, and Sichuan. Specifically, we iteratively remove one province from the above four provinces and re-estimate the model to construct the counterfactuals using the remaining 3 provinces. Figure 10 displays the leave-one-out estimates (gray solid lines) and reproduces Fig. 1 (black solid line and dashed line). All the leave-one-out estimates are above the actual path and surround the counterfactual path during the postintervention period. Therefore, the treatment effects we obtained are robust to the removal of any of the four provinces, although we sacrifice some goodness of fit by excluding one province.

**Fig. 11:**
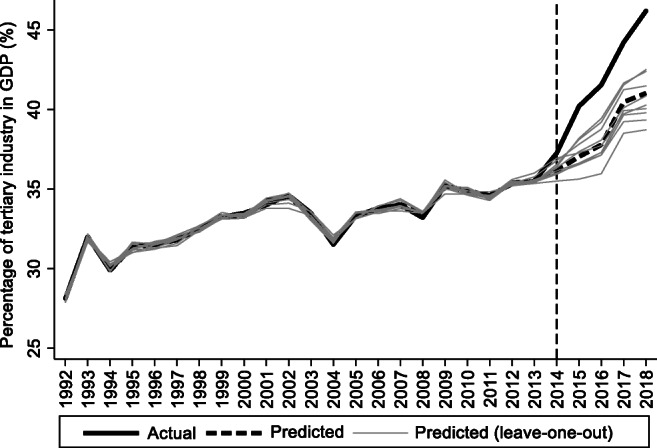
Leave-one-out distribution for the percentage of tertiary industry in GDP

For Hebei’s percentage of tertiary industry in GDP, we use the same approach as applied to the GDP growth rate to test whether the results in Table 6 are sensitive to the leave-one-out design. Figure 11 displays the leave-one-out estimates (gray solid lines) and reproduces Fig. 2 (black solid line and dashed line). Figure 11 shows that the minimum treatment effect for each year corresponds to the highest gray line, and we can see that even the minimum treatment effect is fairly large. Other leave-one-out estimates show either a very similar or a slightly larger effect. Therefore, the treatment effects in Table 6 are robust.

**Fig. 12:**
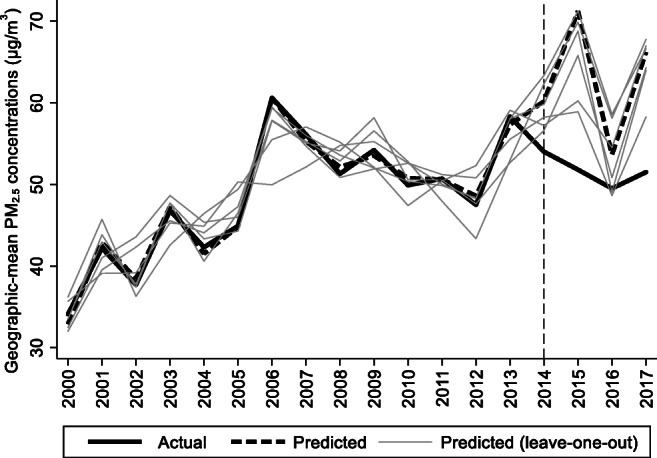
Leave-one-out distribution for the geographic mean PM_2.5_ concentrations

For Hebei’s geographic mean PM_2.5_ concentrations, we also use the leave-one-out approach to test the robustness of the treatment effects in Table 7, with the results shown in Fig. 12. On the whole, the goodness of fit is relatively ideal, with the lowest, 0.71, being the line excluding Fujian, and the highest, 0.92, being the line excluding Jiangxi. Figure 12 shows that in the postintervention period, 6 leave-one-out lines are above the actual path and surround the counterfactual path from 2014 to 2017 on most occasions, except the line excluding Fujian and that excluding Zhejiang in 2016, whose values in 2016 are lower than the actual ones. Therefore, the treatment effects in Table 7 are relatively robust but not as robust as the economic growth rate and industrial structure discussed above. In addition to geographic mean PM_2.5_ concentrations, the Atmospheric Composition Analysis Group also provides population-weighted PM_2.5_ concentrations. Therefore, we also estimate the effects of the Jing-Jin-Ji Strategy on the population-weighted PM_2.5_ concentrations to further verify the environmental effects, which to some extent can be regarded as another robustness test. We will not repeat the estimation process. The treatment effects of the population-weighted PM_2.5_ concentrations, as shown in Table 8, also show significant negative effects, with the largest treatment effect being in 2015 and the second largest being in 2017. Therefore, despite the different data used, the treatment effects resemble each other, which means that the treatment effects for the geographic mean PM_2.5_ concentrations are not incidental but are robust.

**Table 8:** Treatment effects for the population-weighted PM_2.5_ concentrations

Year	Actual	Counterfactual	Treatment effects	
			Point	Interval
2014	78.00	80.78	−2.78	(−4.88, −0.67)
2015	73.80	91.40	−17.60	(−20.31, −14.9)
2016	69.90	76.59	−6.69	(−9.68, −3.69)
2017	69.8	80.89	−11.09	(−14.34, −7.85)

## Conclusion and policy implications

The Jing-Jin-Ji Strategy has attracted immense attention since it was elevated to the status of a national strategy in 2014. This paper examines the causal effects of the Jing-Jin-Ji Strategy on Hebei’s economy and environment by using the PDA. The main findings are that the Jing-Jin-Ji Strategy has significantly upgraded Hebei’s percentage of tertiary industry in GDP and significantly decreased its geographic mean PM_2.5_ concentrations, but it does not appear to have had significant effects on Hebei’s GDP growth rate. The leave-one-out tests demonstrate the robustness of the treatment effects. Therefore, the Jing-Jin-Ji Strategy has made a significant contribution to Hebei’s economy and environment, although the GDP growth rate of Hebei has not increased significantly and has even declined to a certain extent. The primary reason for this lack of effect on GDP growth is that Hebei’s economy has been transitioning from a phase of rapid growth to one of high-quality growth, which means that maximizing the economic growth rate is not the primary goal; rather, the focus is on strengthening the quality of economic development. Therefore, the transformation of the growth model and structural adjustment have been prioritized while ensuring that the economy performs within an appropriate range.

This article makes two contributions to the existing knowledge of the Jing-Jin-Ji Strategy’s treatment effects. First, it analyzes the economic and environmental effects of the Jing-Jin-Ji Strategy on Hebei under the counterfactual framework and reports the counterfactuals for the GDP growth rate, percentage of tertiary industry in GDP, and geographic mean PM_2.5_ concentrations during the postintervention period. Second, it shows that counterfactual analysis is very useful for uncovering the treatment effects of major events, such as national initiatives and natural disasters, on economic and environmental performance.

Our main findings can be useful to policymakers. First, while ensuring the quality of economic development and a sound ecological environment of Hebei, policymakers should help Hebei’s economy develop more rapidly to catch up with Beijing and Tianjin, thereby narrowing the differences within the Jing-Jin-Ji region and enhancing the overall competitiveness of the region. The economic development of Hebei lags far behind that of Beijing and Tianjin, and if this backwardness in Hebei does not fundamentally change, the goals and tasks of the Jing-Jin-Ji Strategy will not be achieved. Second, the treatment effects of the Jing-Jin-Ji Strategy are different at different stages, so it is necessary to assess them dynamically and accurately to adjust the policy direction and ensure its smooth operation.

Some limitations exist in this study. First, Beijing, Tianjin, and Hebei are all treated by the Jing-Jin-Ji Strategy, but only Hebei is examined, and the treatment effects of Hebei cannot be applied to Beijing and Tianjin. Future research can address this by examining the treatment effects of the Jing-Jin-Ji Strategy on Beijing and Tianjin. Second, the research only reveals the economic and environmental effects of the Jing-Jin-Ji Strategy on Hebei, and the transmission mechanisms behind them remain unexplored. Some qualitative (e.g., the process tracing method) or quantitative (e.g., the computable general equilibrium model) approaches could be used in the future to study transmission mechanisms.

## Footnotes

1 For GDP growth rate, the 15 provinces are Shanghai, Zhejiang, Fujian, Jiangxi, Hubei, Hunan, Guangdong, Guangxi, Hainan, Sichuan, Guizhou, Yunnan, Xizang, Qinghai, and Xinjiang; for percentage of tertiary industry in GDP, the 19 provinces are Heilongjiang, Shanghai, Jiangsu, Zhejiang, Anhui, Fujian, Jiangxi, Hubei, Hunan, Guangdong, Guangxi, Hainan, Chongqing, Guizhou, Yunnan, Xizang, Shaanxi, Ningxia, and Xinjiang; for geographic mean PM_2.5_ concentrations, the 18 provinces are Chongqing, Fujian, Guangdong, Guangxi, Guizhou, Hainan, Heilongjiang, Hubei, Hunan, Jiangxi, Jilin, Qinghai, Shanghai, Sichuan, Xinjiang, Xizang, Yunnan, and Zhejiang.
